# Assessment of Cancer Predisposition Syndromes in a National Cohort of Children With a Neoplasm

**DOI:** 10.1001/jamanetworkopen.2022.54157

**Published:** 2023-02-03

**Authors:** Jette J. Bakhuizen, Saskia M. J. Hopman, Machteld I. Bosscha, Charlotte J. Dommering, Marry M. van den Heuvel-Eibrink, Janna A. Hol, Lennart A. Kester, Marco J. Koudijs, Karin P. S. Langenberg, Jan L. C. Loeffen, Jasper van der Lugt, Annette C. Moll, Max M. van Noesel, Stephanie E. Smetsers, Evelien de Vos-Kerkhof, Johannes H. M. Merks, Roland P. Kuiper, Marjolijn C. J. Jongmans

**Affiliations:** 1 Princess Máxima Center for Pediatric Oncology, Utrecht, the Netherlands; 2 Department of Genetics, University Medical Center Utrecht, Utrecht, the Netherlands; 3 Department of Ophthalmology, Amsterdam UMC, Vrije Universiteit Amsterdam, Cancer Center Amsterdam, Amsterdam, the Netherlands; 4 Department of Clinical Genetics, Amsterdam UMC, Vrije Universiteit Amsterdam, Amsterdam, the Netherlands; 5 University Medical Center-Wilhelmina Children’s Hospital, Utrecht, the Netherlands; 6 Division of Imaging and Oncology, University Medical Center Utrecht, Utrecht, the Netherlands; 7 Department of Genetics, University Medical Center Utrecht, Utrecht University, Utrecht, the Netherlands

## Abstract

**Importance:**

To improve diagnostics of cancer predisposition syndromes (CPSs) in children with cancer, it is essential to evaluate the effect of CPS gene sequencing among all children with cancer and compare it with genetic testing based on clinical selection. However, a reliable comparison is difficult because recent reports on a phenotype-first approach in large, unselected childhood cancer cohorts are lacking.

**Objective:**

To describe a national children’s cancer center’s experience in diagnosing CPSs before introducing routine next-generation sequencing.

**Design, Setting, and Participants:**

This retrospective cohort study was conducted at the National Retinoblastoma Treatment Center (Amsterdam, the Netherlands) and the Princess Máxima Center for Pediatric Oncology (Utrecht, Netherlands) and included Dutch pediatric patients with a new diagnosis of neoplasm between June 1, 2018, and December 31, 2019. Follow-up was at least 18 months after neoplasm diagnosis. Data analysis was conducted from July 2021 to February 2022.

**Exposures:**

As part of routine diagnostics, pediatric oncologists and ophthalmologists checked for characteristics of CPSs and selected children for referral to clinical geneticists and genetic testing.

**Main Outcomes and Measures:**

Detected cancer predisposition syndromes.

**Results:**

A total of 824 patients (median [range] age at diagnosis 7.5 [0-18.9] years; 361 girls [44%]) were assessed, including 335 children with a hematological neoplasm (41%) and 489 (59%) with a solid tumor. In 71 of 824 children (8.6%), a CPS was identified, of which most (96%) were identified by a phenotype-driven approach. Down syndrome and neurofibromatosis type 1 were the most common CPSs diagnosed. In 42 of 71 patients (59%), a CPS was identified after these children developed a neoplasm. The specific type of neoplasm was the most frequent indicator for genetic testing, whereas family history played a minor role.

**Conclusions and Relevance:**

In this cohort study of children with a neoplasm, the prevalence of CPSs identified by a phenotype-driven approach was 8.6%. The diagnostic approach for identifying CPSs is currently shifting toward a genotype-first approach. Future studies are needed to determine the diagnostic value, as well as possible disadvantages of CPS gene sequencing among all children with cancer compared with the phenotype-driven approach.

## Key Points

**Question:**

What is the diagnostic yield of phenotype-driven genetic testing in children with cancer?

**Findings:**

In this unselected cohort study of 824 Dutch children with a neoplasm, a cancer predisposition syndrome was found in 71 patients (8.6%), of which most (96%) were identified by a phenotype-driven approach.

**Meaning:**

The diagnostic approach for identifying genetic predisposition in children with cancer is increasingly shifting toward a genotype-first approach; the findings from this phenotype-driven cohort can potentially be used as reference for future genotype-driven studies.

## Teaser

This cohort study examines a Dutch national children’s cancer center’s experience in diagnosing cancer predisposition syndromes before introducing routine next-generation genetic sequencing.

## Introduction

Germline genetic factors play a substantial role in the development of childhood cancer.^1^ Identifying cancer predisposition syndromes (CPSs) in children is important, as it enables surveillance for early detection of second cancers and genetic counseling and testing of relatives. In some CPSs, a modification in treatment is needed.^1,2^ Traditionally, the diagnosis of CPSs in children with cancer is based on clinical suspicion prompting referral to a clinical geneticist. However, the diagnostic approach is increasingly shifting toward a genotype-first approach.^3,4,5^

To improve CPS diagnostics among children with cancer, it is essential to compare the effect of germline sequencing of all childhood cancer predisposition genes vs (targeted) genetic testing based on clinical selection. However, to our knowledge, recent reports on a phenotype-first approach in large, unselected childhood cancer cohorts are lacking. For this, we retrospectively reviewed medical records of children with a new diagnosis of a neoplasm in the Netherlands during a 19-month period (June 2018 to December 2019) before introducing whole-exome sequencing (WES) in the diagnostic work-up. This nationwide, unselected cohort provides insight on the diagnostic yield of a phenotype-first approach and can potentially be used as a reference cohort for genotype-first studies.

## Methods

### Study Design and Study Population

From June 1, 2018, all children with a diagnosis of cancer in the Netherlands were treated centrally at the National Retinoblastoma Treatment Center in Amsterdam (patients with retinoblastoma) or the Princess Máxima Center for Pediatric Oncology in Utrecht (all other children with cancer). Medical records were retrieved for children (age <19 years) who had a new diagnosis of a malignant or benign/borderline neoplasm (except hemophagocytic lymphohistiocytosis and Langerhans cell histiocytosis) between June 1, 2018, and December 31, 2019, who were receiving care in 1 of these 2 centers.

This study was approved by the board of the Princess Máxima Center for Pediatric Oncology. Oral informed consent was obtained for diagnostic procedures. Study-specific informed consent was waived because of the descriptive, observational nature of the study. This study followed, in all relevant parts, the Strengthening the Reporting of Observational Studies in Epidemiology (STROBE) reporting guideline for cohort studies.

### Data Collection

Data collected during the course of care were used for this cohort study. During 2018 and 2019, it was standard practice that children were referred to clinical geneticists on clinical characteristics of CPSs (eg, positive family history for cancer or tumor entity with a strong association with genetic predisposition, such as retinoblastoma) established by the pediatric oncologist or ophthalmologist. During this period, pediatric oncologists and ophthalmologists did not systemically use specific tools or questionnaires to decide which children needed further genetic assessment. The subset of children with Wilms tumors were all offered genetic testing as part of the WES-KidTs study.^6^

Information about baseline characteristics, including age at diagnosis, sex, and neoplasm type, was extracted from electronic medical records at both centers. To evaluate routine CPS diagnostics, medical electronic records were systematically screened for previous medical history and clinical genetic assessment. Reasons for referral were extracted from the referral forms that had been filled in by pediatric oncologists and ophthalmologists and were then categorized into 7 groups (ie, neoplasm type, congenital or other phenotypic anomalies, family history, and child with 2 or more neoplasms [secondary, bilateral, multifocal, or metachronous]), large-scale germline sequencing, genetic tumor analysis, and extensive toxic effects of cancer therapy. A CPS was defined as a clinically and/or molecularly confirmed diagnosis (ie, pathogenic or likely pathogenic variant according to the American College of Medical Genetics and Genomics standards^7^) as concluded by the clinical geneticist who performed the genetic consultation. All patients without a confirmed CPS were considered no CPS identified. For patients with a confirmed CPS, additional data on the diagnostic process were collected, including timing of genetic testing and performed genetic tests. Medical electronic records were checked at least 18 months after neoplasm diagnosis.

### Statistical Analysis

Descriptive statistics were presented as median (range) for continuous variables and as frequencies (percentage) for categorical variables. Statistical analyses were performed using Microsoft Excel. Data were analyzed from July 2021 to February 2022.

## Results

### Description of the Unselected Nationwide Cohort

From June 2018 to January 2020, 824 children (median [range] age at diagnosis, 7.5 [0.0-18.9] years; 361 girls [44%]) had a new diagnosis of cancer in the Netherlands. Cohort details are summarized in the Table. The cohort included 335 children with a hematologic neoplasm (40.7%), 309 children with a non–central nervous system solid tumor (37.5%), and 180 children with a solid tumor of the central nervous system (21.8%).

**Table.** Patient Characteristics

Characteristic	No. (%)
Sex	
Female	361 (43.8)
Male	463 (56.2)
Age at diagnosis of neoplasm, y	
0-5	368 (44.7)
6-10	146 (17.7)
11-15	216 (26.2)
16-18	94 (11.4)
Diagnosis	
Hematologic neoplasms	335 (40.7)
Acute lymphoblastic leukemia	175 (21.2)
Hodgkin lymphoma	64 (7.8)
Non-Hodgkin lymphoma	42 (5.1)
AML/CML/other myeloid leukemia	41 (5.0)
Myelodysplastic syndrome/aplastic anemia	10 (1.2)
Other	3 (0.4)
Non-CNS solid tumors	309 (37.5)
Neuroblastoma	47 (5.7)
Wilms tumor	43 (5.2)
Osteosarcoma	34 (4.1)
Rhabdomyosarcoma	32 (3.9)
Germcell tumor	35 (4.2)
Ewing (like) sarcoma	27 (3.3)
Fibrosarcomas and other fibrous neoplasms	16 (1.9)
Retinoblastoma	14 (1.7)
Sarcoma other	10 (1.2)
Liver tumor	9 (1.1)
Melanoma	7 (0.8)
Kidney tumor other than Wilms tumor	7 (0.8)
Extra kidney rhabdoid tumor	4 (0.5)
Other	24 (2.9)
CNS tumors	180 (21.8)
Low-grade glioma, WHO grade 1-2	78 (9.5)
High-grade glioma, WHO grade 3-4	28 (3.4)
Medulloblastoma	29 (3.5)
Ependymoma	14 (1.7)
Germcell tumor	12 (1.5)
Craniopharyngioma	6 (0.7)
Rhabdoid tumor	3 (0.4)
Schwannoma	3 (0.4)
Other	7 (0.8)

Abbreviations: AML, acute myeloid leukemia; CML, chronic myeloid leukemia; CNS, central nervous system; WHO, World Health Organization.

One hundred eighty-seven of 824 children (23%) were referred to a clinical geneticist following neoplasm diagnosis (eFigure in Supplement 1). After clinical genetic assessment, 166 of the 187 patients (88.8%) were offered (targeted) genetic testing. In the remaining 21 patients, no genetic testing was performed because the clinical constellation appeared unlikely to be associated with a known CPS.

### Patients Identified With a CPS

**Figure 1.**
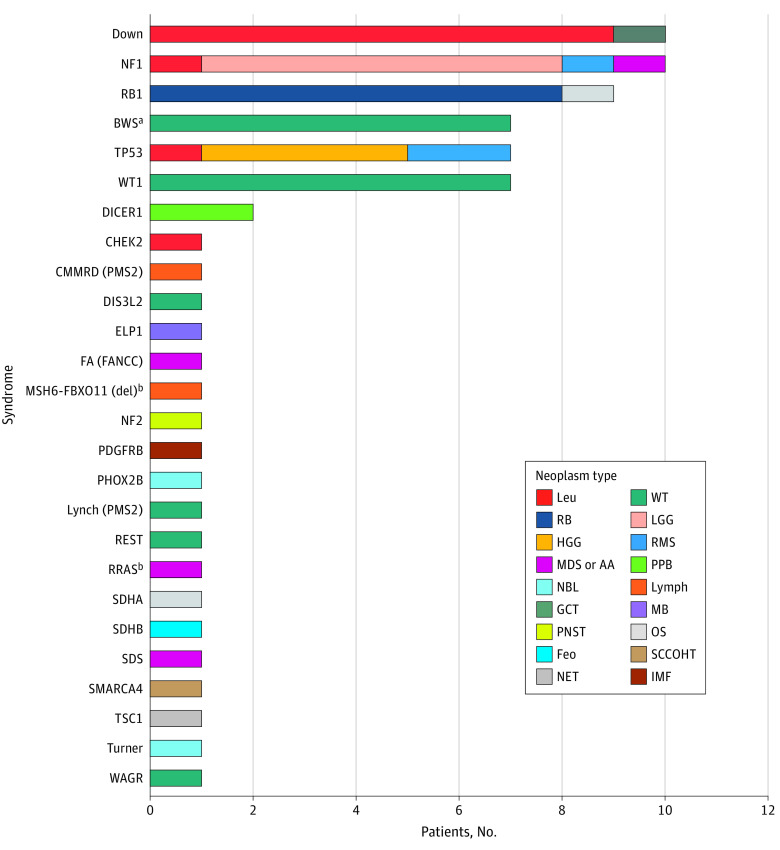
Identification of 71 Cancer Predisposition Syndromes in a National, Unselected Cohort of 824 Children With a Neoplasm AA indicates aplastic anemia; BWS, Beckwith-Wiedemann spectrum; CMMRD, constitutional mismatch repair deficiency; FA, Fanconi anemia; feo, feochromocytoma; GCT, germ cell tumor; HGG, high-grade glioma; IMF, infantile myofibromatosis; leu, leukemia; LGG, low-grade glioma; lymph, lymphoma; MB, medulloblastoma; MDS, myelodysplastic syndrome; NBL, neuroblastoma; NET, neuroendocrine tumor; NF1, neurofibromatosis type 1; OS, osteosarcoma; PNST, peripheral nerve sheath tumor; PPB, pleuropulmonary blastoma; RB, retinoblastoma; RMS, rhabdomyosarcoma; SCCOHT, small-cell carcinoma of the ovary hypercalcemic type; SDS Shwachman-Diamond syndrome; TSC, tuberous sclerosis complex; WT, Wilms tumor. ^a^Including molecular diagnosis for BWS based on methylation-specific MLPA nontumor kidney tissue.^6^ ^b^Patients were described in case reports.^8,9^

In 71 of 824 patients (8.6%) a CPS was identified, including 26 different syndromes (Figure 1). In 68 patients (96%), the CPS was identified by a phenotype-first approach. In 3 patients, the CPS was identified by performing WES in a genotype-first approach as part of a precision medicine program or tumor diagnostic workup. The prevalence of CPSs ranged from 5.4% for patients with hematologic neoplasms to 7.8% for central nervous system tumors, and 12.6% for solid tumors. Down syndrome (n = 10) and neurofibromatosis type 1 (n = 10) were the most common CPSs diagnosed. Most CPSs were molecularly confirmed (69 of 71 cases). In 66 patients (93%), the identified CPS explained neoplasm development. In 2 of these patients, who both had a diagnosis of a high-grade glioma and germline *TP53* pathogenic variant, the CPS was not identified by the phenotype-first approach, but rather detected by performing WES as part of a precision medicine program or tumor diagnostic work-up. In 5 patients, germline pathogenic variants were identified in genes for which there are currently no evidence for a causal role in the development of neoplasms in these children (ie, *CHEK2* in acute myeloid leukemia, heterozygous *PMS2* in Wilms tumor, *SDHA* in osteosarcoma, *MSH6::FBXO11* in diffuse large B-cell lymphoma,^8^ and Turner syndrome in neuroblastoma).

### Diagnostic Process of Identified CPSs

**Figure 2.**
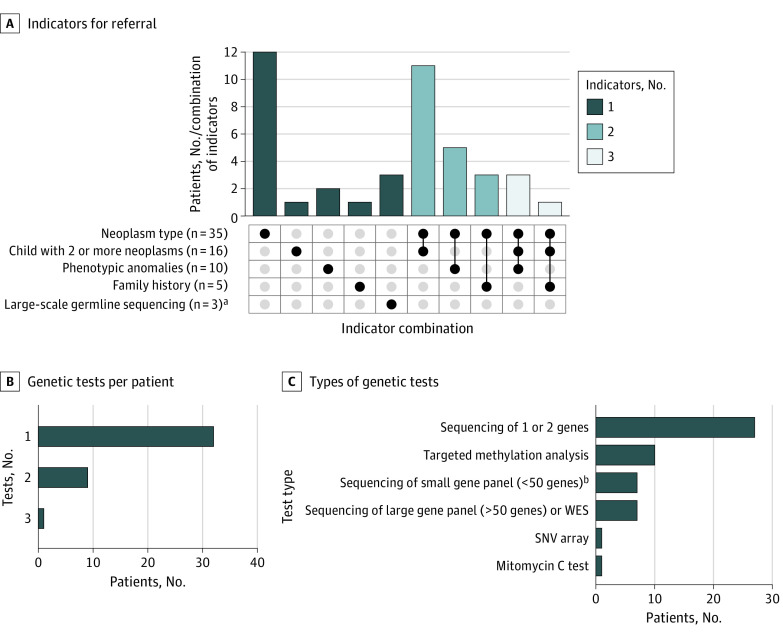
Diagnostic Process of Identified Cancer Predisposition Syndromes After Neoplasm Development A, Specific indicators that initiated referral for clinical genetic assessment in 42 patients for whom genetic predisposition was identified after they had developed a neoplasm. The total number of patients per indicator for referral are noted in the column labels. Each column corresponds to a specific combination, and bar charts on top show the number of patients per combination. The filled-in dots show which indicator for referral is part of a combination. The feature “child with 2 or more neoplasms” is defined as the presence of bilateral, multifocal, or metachronous primary neoplasms. B, Number of genetic tests that were performed per patient. C, Type of genetic tests that were performed. SNV, single-nucleotide variation; WES, whole-exome sequencing. ^a^Part of the tumor diagnostic workup or precision medicine study. ^b^Including a kidney tumor predisposition gene panel that was offered to all children with Wilms tumors as part of the WES-KidTs study.^6^

Twenty-nine of the 71 patients with a CPS (41%) had received a diagnosis of this syndrome before they developed a neoplasm. Most of these children had specific phenotypic features that allowed for a clinical diagnosis (eg, signs of Down syndrome or neurofibromatosis type 1). In patients in whom genetic predisposition was identified after they had developed a neoplasm, the specific type of neoplasm was the most frequent indicator (35 of 42 patients [83.3%]) for referral to a clinical geneticist (Figure 2A), although often in combination with other features. In most patients, the genetic predisposition was revealed by performing targeted genetic testing, being either sequencing of 1 or 2 genes or targeted methylation analysis (Figure 2, B and C).

## Discussion

In this national unselected cohort study of 824 children with a neoplasm, 8.6% received a diagnosis of a CPS, of which most (96%) were identified by a phenotype-driven approach. The tumor entity was the most frequent reason for genetic testing. In contrast to many adult-onset CPSs, family history only played a limited role in recognition of the underlying predisposition. A possible explanation for this is the high contribution of de novo variants. In this cohort, 37 of 58 patients (64%) with sufficient information about inheritance mode carried a de novo pathogenic variant. In addition, 3 patients received a diagnosis of an autosomal-recessive CPS.

The prevalence of CPSs identified in the unselected cohort was similar to the 7% to 12% that was reported in earlier genotype-based studies.^3,10,11^ In a recent prospective sequencing study, a higher prevalence of germline cancer-predisposing variants was reported (18%), but heterozygous variants in genes associated with autosomal recessive inherited CPSs were included.^5^ Whereas the mostly phenotype-driven diagnosis of CPSs in the current unselected cohort revealed a CPS prevalence that was similar to that of earlier genotype-based studies, the spectrum of CPS diagnoses is different between the 2 approaches. For example, Down syndrome, often excluded from these large sequence studies, was found to be the most common CPS in the current cohort, whereas relatively few children were identified with a germline *TP53* pathogenic variant. The higher prevalence of certain CPSs, like Li-Fraumeni syndrome, in genotype-based studies is partly due to the biased selection of cancer types typically found in Li-Fraumeni syndrome (such as adrenocortical tumors and hypodiploid acute lymphocytic leukemia).^3,10^ However, a phenotype-driven approach is also associated with an underestimation of CPSs with less prominent clinical features.

We hypothesize that identification of CPSs in children with cancer can be optimized by combining comprehensive phenotyping (clinical history and examination) with systematic genetic tests, even in patients without specific clinical features. This is illustrated in a recent study in patients with Wilms tumors in which a comprehensive and stepwise approach of diagnostic genetic testing and research-based WES analysis was performed.^6^ Whether such a combined approach is also useful in children with other types of cancer has to be proven. To our knowledge, to date, only 2 studies have combined comprehensive clinical data and WES/whole-genome sequencing in an unselected cohort of pediatric patients.^4,12^ Pathogenic germline variants were found in 6.9% and 14.6% of patients, but relatively few children were included.

### Strengths and Limitations

A strength of this study is that it presents an unselected cohort of pediatric patients with cancer with a very recent CPS diagnosis using a phenotype-driven approach. As such, this study can potentially be used as a reference cohort for future genotype-driven or combined phenotype-genotype–driven studies. The study took place in centers with specific expertise in childhood CPSs, and CPS diagnoses may be higher because of this expertise compared with other (smaller) centers.

A limitation of this study is the lack of a structured evaluation of family history and clinical features. The data represent a real-life routine care setting where pediatric oncologists and ophthalmologists checked for characteristics of CPSs; therefore, it is likely that not all children were systematically examined. The use of a specific tool to identify patients at risk of a CPS, such as the McGill Interactive Pediatric OncoGenetic Guidelines, may increase the detection of CPSs by a phenotype-based approach.^13^ In addition, to specify the diagnostic value of the phenotype-based approach, a direct comparison between targeted genetic testing based on clinical selection and CPS gene sequencing in all children with cancer is needed.

## Conclusions

In this cohort study of children with a neoplasm, the prevalence of CPSs identified by a phenotype-driven approach was 8.6%. Whereas the CPS prevalence in this cohort was similar to that in earlier genotype-based studies, the spectrum of CPS was different. To determine whether CPS genes sequencing among all children with cancer can serve as a replacement or add-on test, the diagnostic yield, as well as other relevant aspects, such as costs and the number of unsolicited findings, should be compared with that of the classical phenotype-driven diagnostic approach.

## Notes

Supplement 1. **eFigure.** Flowchart of clinical genetic assessment and genetic testing in patients with a neoplasm

Supplement 2.
Data sharing statement
