# Supplementary material for: Assessment of Cancer Predisposition Syndromes in a National Cohort of Children With a Neoplasm
Source: JAMA Netw Open. 2023 Feb 3;6(2):e2254157. doi: 10.1001/jamanetworkopen.2022.54157 (PMC9898819; doi:10.1001/jamanetworkopen.2022.54157)
Supplement: Supplement 1. — eFigure. Flowchart of clinical genetic assessment and genetic testing in patients with a neoplasm [file jamanetwopen-e2254157-s001.pdf]

Supplemental Online Content

Bakhuizen JJ, Hopman SMJ, Bosscha MI, et al. Assesment of cancer predisposition syndromes in a national cohort of children with a neoplasm. *JAMA Network Open*. 2023;6(2):e2254157. doi:10.1001/jamanetworkopen.2022.54157

eFigure. Flowchart of clinical genetic assessment and genetic testing in patients with a neoplasm

This supplemental material has been provided by the authors to give readers additional information about their work.

© 2023 Bakhuizen JJ et al. *JAMA Network Open.*

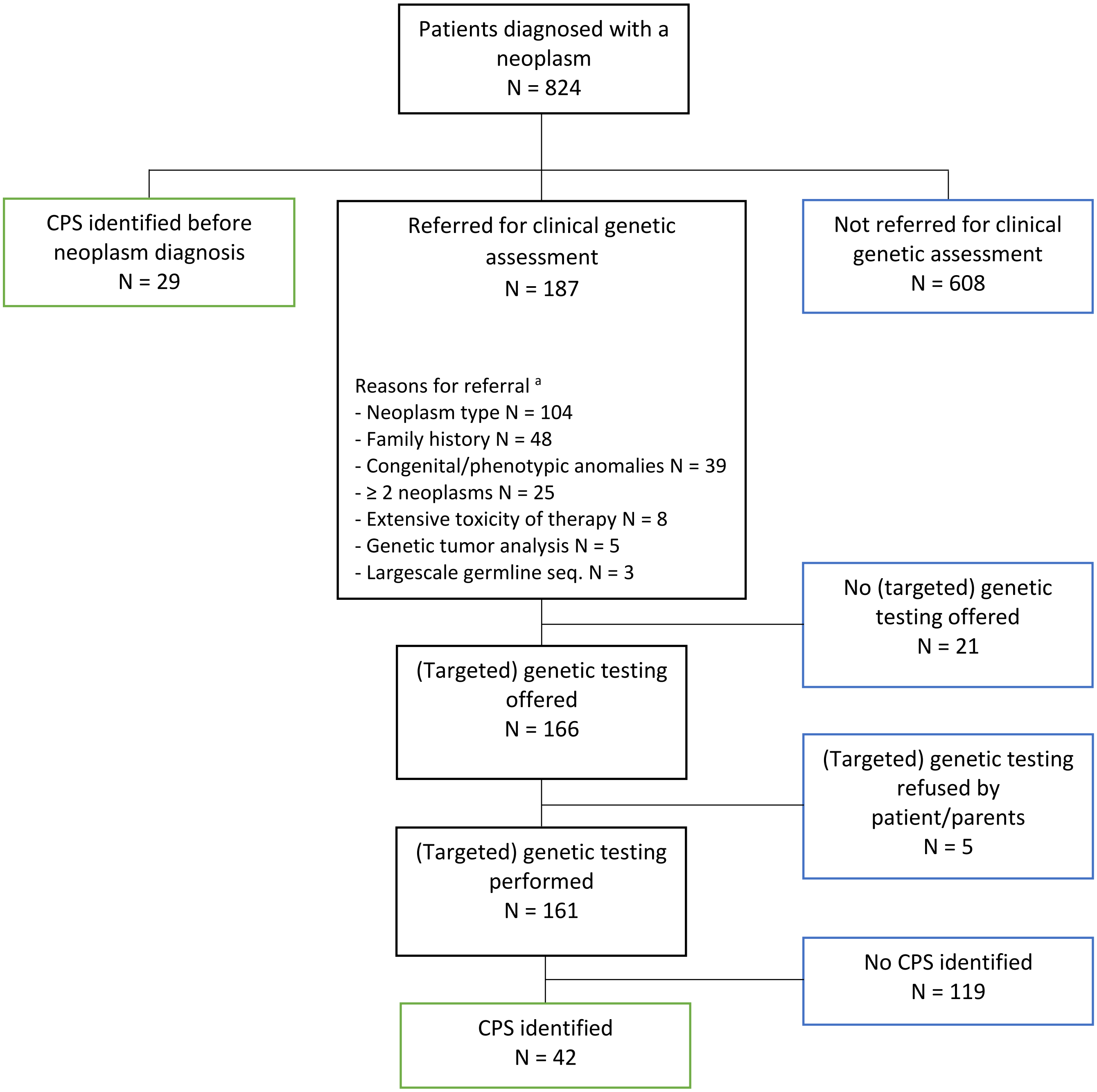

eFigure. Flowchart of clinical genetic assessment and genetic testing in patients with a neoplasm

[a] Reasons for referral were based on the referral forms that had been filled in by pediatric oncologists and ophthalmologists. The total number of reasons for referral exceeds the number of patients that were referred for clinical genetic assessment because patients could have been referred based on more than one reason.

© 2023 Bakhuizen JJ et al. *JAMA Network Open.*
