# Supplementary material for: Assessment of Cancer Predisposition Syndromes in a National Cohort of Children With a Neoplasm
Source: JAMA Netw Open. 2023 Feb 3;6(2):e2254157. doi: 10.1001/jamanetworkopen.2022.54157 (PMC9898819; doi:10.1001/jamanetworkopen.2022.54157)
Supplement: Supplement 2. — Data sharing statement [file jamanetwopen-e2254157-s002.pdf]

Data Sharing Statement

Bakhuizen. Assessment of Cancer Predisposition Syndromes in a National Cohort of Children With a Neoplasm. *JAMA Netw Open*. Published February 03, 2023.
doi:10.1001/jamanetworkopen.2022.54157

Data
Data available: No
